# Injury and Social Correlates among in-School Adolescents in Four Southeast Asian Countries

**DOI:** 10.3390/ijerph9082851

**Published:** 2012-08-13

**Authors:** Karl Peltzer, Supa Pengpid

**Affiliations:** 1 HIV/STI and TB (HAST) Research Programme, Human Sciences Research Council, 134 Pretorius Street, 0002 Pretoria, South Africa; 2 Department of Psychology, University of Limpopo, Turfloof Campus, Sovenga, 0727 Limpopo, South Africa; 3 Department of Health System Management and Policy, University of Limpopo, Ga-Rankuwa Campus, Medunsa, 0204 Pretoria, South Africa; Email: supaprom@yahoo.com

**Keywords:** injury, social correlates, school children, Indonesia, Myanmar, Sri Lanka, Thailand

## Abstract

The aim of this study was to determine estimates of the prevalence and social correlates of injury among adolescents in four Southeast Asian countries. Cross-sectional national data from the Global School-based Health Survey (GSHS) included 9,333 students at the ages from 13 to 15 years inclusive from Indonesia, Myanmar, Sri Lanka and Thailand is chosen by a two-stage cluster sample design to represent all students in grades 6, 7, 8, 9, and 10 in each country. The percentage of adolescents reporting one or more serious injuries within the past 12 months was 42.2% for all countries, ranging from 27.0% in Myanmar to 46.8% in Thailand. By major activity, “fall” (14.6%) was the leading external cause of injury, followed by playing or training for a sport (9.9%) and vehicle accident (6.1%). In multivariate regression analysis Thailand and Indonesia, being male, substance use (smoking and drinking alcohol) and psychological distress were associated with annual injury prevalence. Risk factors of substance use and psychological distress should be considered in an integrated approach to injury etiology in planning injury prevention and safety promotion activities among school children.

## 1. Introduction

Globally, 98% of all childhood unintentional injuries occur in low and middle income countries [1]. Unintentional injuries are a major cause of death and disability among children [2]. An analysis of the 1990 Global Burden of Disease study found that the childhood injury rate was the highest in Africa and South Asia [3]. The annual prevalence of serious injuries was 68.2% among 13 to 15 year-olds in six African countries [4] and among 11-, 13- and 15-year old youth in 11 industrialised countries was 41.3% [5]. In a study among school children in Kamphaeng Phet Province in Thailand 66% reported at least one injury in the previous year and the leading categories of non-fatal injuries were: animal bite, puncture wound, burn, near-drowning, fall from a height [6]. Among young people (10–24 years) injury is the most common (43%) cause of death in the World Health Organization (WHO) Southeast Asia (SEA) region [7]. These include traffic accidents, violence, fire-related incidents, and drowning [8]. Community-based studies have shown the extent of the problem of unintentional injuries in children and young adolescents, with rates of non-fatal injuries of 14 per 1,000 in Thailand (1–17 years) and 220 in Sri Lanka (0–19 years) [9]. In a pilot study of childhood injuries in Yangon General Hospital, 2003, in Myanmar, 30.8% of total injured patients reported were children under 15 years of age. Various types of “falls” (66%) were identified as the major cause of child injury followed by road traffic accidents (22%) [10]. A study among children (less than 13 years old) seeking hospital treatment in Sri Lanka found that unintentional injuries within the home and on the road comprised 56% and 8%, respectively of all causes of injury [10]. In a community-based study in the Galle district, Southern Sri Lanka, 1.4% and 1.1% non-fatal injuries in the last 30 days were found among 10 to 14 year-olds and 15 to 19 year-olds, respectively [11]. The national injury surveillance system in Thailand found that transport accidents ranked first (39.2%), followed by accidental falls (27.6%) and exposure to inanimate forces (16.4%) for severe injury in children less than 15 years in 2005 [10]. 

“The etiology of youth injury involves a complex interplay between human and environmental factors” [12]. Various studies have identified multiple risk behaviour including substance use, bullying and psychological distress [13,14,15,16,17], obesity [18], low socioeconomic status [12,19], male gender [8,15], home and school environment [16] to be associated with injury risk. 

There is lack of national data regarding injury and its social correlates among in-school adolescents in Southeast Asia. Therefore, the aim of this study was to determine estimates of the prevalence and social correlates of injury among adolescents in four Southeast Asian countries. 

## 2. Methodology

### 2.1. Description of Survey and Study Population

This study involved secondary analysis of existing data from the Global School-Based Health Survey (GSHS) from four Southeast Asian countries (Indonesia, Myanmar, Sri Lanka and Thailand). Details and data of the GSHS can be accessed at http://www.who.int/chp/gshs/methodology/ en/index.html. The aim of the GSHS is to collect data from students of age 13 to 15 years inclusive. The GSHS is a school-based survey of students in grades 6, 7, 8, 9, and 10. These classes were selected because they contained the majority of 13 years to 15 years old school adolescents. A two-stage cluster sample design was used to collect data to represent all students in grades 6, 7, 8, 9, and 10 in the country. At the first stage of sampling, schools were selected with probability proportional to their reported enrollment size. In the second stage, classes in the selected schools were randomly selected and all students in selected classes were eligible to participate irrespective of their actual ages. Students completed the self-administered questionnaire during one classroom period under the supervision of trained survey administrators and recorded their responses to each question on an answer sheet suitable for computerized scanning. 

### 2.2. Measures

The GSHS 10 core questionnaire modules address the leading causes of morbidity and mortality among children and adults world wide: tobacco, alcohol and other drug use; dietary behaviours; hygiene; mental health; physical activity; sexual behaviours that contribute to HIV infection, other sexually-transmitted infections, and unintended pregnancy; unintentional injuries and violence; hygiene; protective factors and respondent demographics [20]. One study assessed the validity of the GSHS questionnaire and found adequate retest reliability of GSHS content adapted for ethnic Fijian girls for assessing several risk behaviours [21].

## 3. Results and Discussion

### 3.1. Sample

The sample included 9,333 students at the ages from 13 to 15 years from Southeast Asian countries; there were slightly more female (50.4%) than male students (49.6%) and the majority of the students (76.2%) were attending school grades 8 or 9. Data from the different countries had been selected in 2007 or 2008 (see Table 1). The overall response rate, a product of school and student response rates, varied from 89% in Sri Lanka to 95% in Myanmar.

**Table 1:** Sample response rate and age distribution of students surveyed; GSHS 2007–2008.

Country	Survey sample N	Survey year	Overall response rate % *	Age groups in years (%)					Boys in final sample %	Mean age of final sample	Net primary school enrolment rate % [22,23]	
				13 years	14 years		15 years				Male	Female
1. Indonesia	2,867	2008	93	1,072 (33.2)		1,253 (45.2)		542 (21.6)	49.5	13.9	97	94
2. Myanmar	1,983	2007	95	585 (37.1)		628 (34.3)		770 (28.6)	50.0	13.9	90	91
3. Sri Lanka	2,260	2007	89	894 (38.9)		844 (37.3)		522 (23.8)	50.4	13.8	99	100
4. Thailand	2,223	2008	93	841 (37.1)		871 (36.2)		511 (26.7)	49.2	13.9	91	89

* Overall response rate, the product of school and the student response rate, refers to the entire sample including those students outside the targeted age range of 13 to 15 years.

**Table 2:** Annual prevalence of injury events by sex and country in percent (95%CI).

	**Total**	**Boys**	**Girls**	**Indonesia**	**Myanmar**	**Sri Lanka**	**Thailand**
INJURY (in the past 12 months)	42.2 (39.7–44.8)	50.5 (31.8–36.3)	34.3 (31.8–36.9)	45.9 (41.7–50.1)	27.0 (21.9–32.0)	37.2 (32.0–42.5)	46.8 (42.3–51.0)
Injured once	25.8 (24.0–27.5)	29.1 (27.1–31.1)	22.5 (20.3–24.8)	28.1 (24.7–31.5)	19.3 (15.0–23.7)	23.6 (20.3–26.8)	26.4 (23.9–28.9)
Injured more than once	16.4 (14.9–18.1)	21.4 (19.3–23.5)	11.8 (10.5–13.1)	17.8 (15.1–20.5)	7.7 (5.5–9.9)	13.7 (10.9–16.5)	20.3 (16.9–23.8)
ACTIVITY (of most serious injury)							
Playing or training for a sport	9.9 (8.9–10.9)	14.6 (13.2–16.0)	5.5 (4.4–6.5)	10.0 (8.7–11.4)	5.9 (4.3–7.6)	11.2 (8.0–14.4)	11.3 (9.6–13.1)
Walking or running, but not as part of playing or training for a sport	5.1 (4.4–5.8)	5.6 (4.7–6.6)	4.6 (3.7–5.5)	6.1 (5.1–7.1)	2.8 (1.8–3.7)	6.2 (4.8–7.6)	4.4 (3.3–5.6)
Riding a bicycle or scooter	3.5 (2.9–4.2)	4.7 (3.9–5.6)	2.4 (1.7–3.1)	2.7 (1.6–3.8)	4.3 (2.9–5.7)	5.7 (4.4–6.9)	3.5 (2.3–4.7)
Riding or driving in a car, or other motor vehicle	3.7 (3.1–4.4)	4.3 (3.5–5.1)	3.2 (2.3–4.0)	3.2 (2.1–4.3)	0.3 (0.1–0.6)	0.5 (0.1–0.9)	7.8 (6.3–9.4)
Doing any paid or unpaid work, including housework, yard work, *etc*.	3.1 (2.5–3.6)	2.8(2.0–3.6)	3.3 (2.5–4.2)	3.0 (2.1–3.9)	3.0 (1.9–4.1)	4.7 (3.8–5.6)	2.6 (1.7–3.5)
Nothing	5.4 (4.7–6.1)	5.6 (4.4–6.8)	5.2 (4.1–6.3)	10.2 (8.5–11.8)	1.1 (0.7–1.5)	1.5 (1.2–1.9)	1.9 (1.2–2.5)
Something else	4.6 (4.0–5.1)	4.5 (3.6–5.3)	4.7 (3.8–5.6)	4.8 (3.9–5.7)	2.1 (1.2–3.1)	2.8 (1.7–4.0)	6.2 (5.0–7.4)
CAUSE (of most serious injury)							
I was in a motor vehicle accident or hit by a motor vehicle	6.1 (5.3–6.9)	7.8 (6.8–8.8)	4.5 (3.5–5.6)	7.3 (5.8–8.8)	2.0 (1.2–2.7)	2.9 (2.0–3.8)	8.0 (6.4–9.7)
I fell something fell on me or hit me	14.6 (13.2–15.9)	17.2 (15.6–18.8)	12.1 (10.6–13.5)	17.7 (15.6–19.8)	8.5 (6.8–10.2)	16.2 (12.4–20.0)	12.3 (10.3–14.3)
	3.1 (2.6–3.6)	3.3 (2.7–3.9)	2.9 (2.3–3.6)	2.6 (1.9–3.3)	3.4 (2.2–4.6)	4.1 (3.2–5.0)	3.4 (2.3–4.4)
I was fighting with someone	1.6 (1.2–2.0)	2.6 (2.0–3.3)	0.6 (0.4–0.9)	1.3 (0.6–1.9)	1.0 (0.4–1.7)	0.9 (0.4–1.3)	2.8 (2.0–3.7)
I was attacked or assaulted or abused by someone	0.8 (0.5–1.0)	0.8 (0.5–1.1)	0.7 (0.3–1.0)	1.0 (0.6–1.4)	0.3 (0.0–0.6)	0.8 (0.3–1.2)	0.7 (0.3–1.0)
I was in a fire or too near a flame or something hot	0.5 (0.2–0.9)	0.4 (0.2–0.6)	0.7 (0.1–1.2)	0.7 (1.7–1.2)	0.8 (0.1–1.4)	0.3 (0.0–0.6)	0.3 (0.0–0.6)
Something else caused my injury	8.1 (7.4–8.8)	9.5 (8.6–10.4)	6.7 (5.8–7.7)	9.9 (8.7–11.1)	2.8 (1.8–3.9)	6.1 (4.9–7.3)	9.0 (7.4–10.5)
HOW INJURY HAPPENED (of most serious injury)							
I hurt myself by accident	20.7 (19.2–22.3)	23.7 (21.8–25.7)	17.9 (16.0–19.9)	22.4 (19.5–25.3)	14.9 (12.0–17.8)	20.2 (16.8–23.7)	21.3 (18.6–24.0)
Someone else hurt me by accident	10.5 (9.4–11.5)	13.0 (11.5–14.5)	8.1 (7.0–9.3)	13.3 (11.5–15.1)	4.2 (2.9–5.5)	7.1 (5.5–8.8)	10.9 (9.2–12.7)
I hurt myself on purpose	1.2 (0.9–1.4)	1.7 (1.2–2.2)	0.7 (0.4–0.9)	0.8 (0.3–1.2)	1.2 (0.7–1.8)	0.9 (0.4–1.3)	1.9 (1.2–2.5)
Someone else hurt me on purpose	2.1 (1.6–2.7)	2.8 (1.8–3.8)	1.4 (1.0–1.8)	2.6 (1.4–3.7)	0.9 (0.5–1.3)	1.7 (0.8–2.5)	2.3 (1.5–3.1)
TYPE OF INJURY (of most serious injury)							
I had a broken bone or a dislocated joint	10.1 (8.8–11.4)	14.1 (12.3–15.9)	6.2 (5.1–7.3)	16.0 (13.5–18.4)	5.1 (3.6–6.6)	6.8 (5.3–8.2)	5.2 (4.3–6.2)
I had a cut, puncture, or stab wound	5.1 (4.4–5.9)	6.6 (5.7–7.5)	3.7 (2.9–4.6)	3.5 (2.5–4.5)	7.7 (5.9–9.5)	9.4 (7.7–11.0)	4.3 (2.8–5.7)
I had a concussion or other head or neck injury, was knocked out, or could not breath	1.8 (1.4–2.1)	1.8 (1.4–2.3)	1.7 (1.2–2.2)	1.4 (0.9–1.9)	1.1 (0.5–1.8)	2.6 (1.9–3.3)	2.2 (1.5–2.9)
I had a gunshot wound	0.4 (0.2–0.6)	0.5 (0.2–0.8)	2.9 (0.1–4.9)	0.3 (0.0–2.1)	0.2 (0.1–0.4)	0.5 (0.2–0.8)	0.7 (0.2–1.2)
I had a bad burn	1.3 (0.9–1.7)	1.4 (0.9–1.9)	1.3 (0.8–1.7)	1.4 (0.7–2.1)	0.4 (0.0–0.8)	0.9 (0.6–1.3)	1.8 (1.1–2.5)
I lost all or part of a foot, leg, hand, or arm	0.3 (0.1–0.5)	0.4 (0.1–0.7)	0.2 (0.0–0.5)	0.5 (0.2–0.8)	0.1 (0.0–0.2)	0.4 (0.1–0.9)	0.2 (0.0–0.4)
Something else happened to me	14.2 (13.2–15.3)	15.2 (13.7–16.6)	13.4 (12.0–14.8)	15.9 (14.0–17.7)	4.2 (2.7–5.7)	9.2 (8.0–10.3)	19.3 (17.2–21.4)

**Table 3:** Logistic regression analysis for association between risk behaviours and injury (overall analysis for all injury types) and between the number of risk behaviours and restricted analysis by type/context of injury.

	All injuries		Fall injuries	Sports injuries	Motor vehicle injuries	Fighting injuries
	Odds ratio (95% CI)					
Variables	Crude	Adjusted	Adjusted	Adjusted	Adjusted	Adjusted
*Country*						
Indonesia	1	1	1	1	1	1
Myanmar	0.44 (0.31–0.61) ***	0.48 (0.34–0.69) ***	0.50 (0.39–0.64) ***	0.55 (0.40–0.76) ***	0.22 (0.13–0.37) ***	1.35 (0.59–3.09)
Sri Lanka	0.70 (0.52–0.93) *	0.68 (0.52–0.90) **	0.90 (0.66–1.24)	1.15 (0.83–1.59)	0.38 (0.26–0.55) ***	0.69 (0.34–1.40)
Thailand	1.04 (0.82–1.31)	1.24 (0.91–1.69)	0.63 (0.47–0.84) **	1.24 (0.91–1.69)	0.96 (0.67–1.37)	2.38 (1.60–3.59) ***
*Age*						
13	1	1	1	1	1	1
14	1.12 (0.97–1.30)	1.03 (0.79–1.14)	1.01 (0.89–1.14)	1.19 (0.87–1.61)	1.61 (1.06–2.41) *	0.69 (0.37–1.30)
15 years	1.04 (0.86–1.26)	1.00 (0.72–1.12)	0.87 (0.69–1.09)	1.25 (0.94–1.67)	1.68 (1.09–2.60) *	0.98 (0.63–1.52)
*Gender*						
Female	1	1	1	1	1	1
Male	1.95 (1.72–2.21) ***	1.64 (1.38–1.95) ***	1.29 (1.08–1.55) **	3.38 (2.55–4.47) ***	1.82 (1.39–2.39) ***	4.27 (2.35–7.74) ***
Hunger (4.6%)	1.67 (1.14–2.44) **	1.31 (0.79–2.17)	1.11 (0.66–1.84)	1.30 (0.74–2.28)	1.22 (0.60–2.49)	0.72 (0.17–3.10)
Current smoking (8.7%)	3.53 (2.67–4.67) ***	2.01 (1.39–2.91) ***	1.29 (0.98–1.69)	1.17 (0.81–1.60)	1.17 (075–1.80)	3.13 (1.54–6.35) **
Current drinking (6.5%)	3.48 (2.67–4.53) ***	1.80 (1.20–2.69) **	1.13 (0.76–1.67)	1.03 (0.64–1.65)	1.96 (1.27–3.03) **	2.76 (1.02–7.45) *
Ever illicit drugs (2.4%)	6.20 (3.83–10.01) ***	1.51 (0.79–2.89)	1.05 (0.40–2.77)	1.07 (0.44–2.60)	0.37 (0.14–1.03)	0.22 (0.05–1.00)
*Psychological distress*						
Zero (70.6%)	1	1	1	1	1	1
One (20.7%)	1.99 (1.72–2.29) ***	1.93 (1.58–2.34) ***	1.31 (1.04–1.66) *	1.45 (1.08–1.94) *	1.34 (0.92–1.95)	2.60 (1.35–5.04) **
Two or more (8.7%)	2.71 (2.07–3.53) ***	2.43 (1.77–3.32) ***	1.06 (0.70–1.61)	1.14 (0.81–1.60)	1.46 (0.95–2.25)	4.49 (2.55–8.24) ***

*** *P* < 0.0001, ** *P* < 0.01, * *P* < 0.05

### 3.2. Descriptive Results

The percentage of adolescents reporting one or more serious injuries within the past 12 months was 42.2% for all countries, ranging from 27.0% in Myanmar to 46.8% in Thailand, and it has been slightly more often in boys (50.5%) than girls (34.3%) in all countries. Estimates of adolescents reporting a single injury were less variable, ranging from 19.3% in Myanmar to 28.1% in Indonesia, while similar differences in prevalence estimates by country were found in the number of adolescents reporting multiple injuries, ranging from 7.7% to 20.3% in Myanmar and Thailand, respectively. By major activity of all survey participants, “fall” (14.6%) was the leading external cause of injury, followed by playing or training for a sport (9.9%), vehicle accident (6.1%), walking or running (5.1%), riding a bicycle or scooter (3.7%), fighting with someone (1.6%), and attacked or assaulted or abused by someone (0.8%). The majority of all surveyed adolescents (20.7%) indicated that they had hurt themselves by accident. The injury sustained by most students of all surveyed involved broken bone/dislocated joint (10.1%), followed by a cut, puncture, stab wound (5.1%), concussion/head injury (1.8%) and burn injury (1.3%) (see Table 2).

### 3.3. Associations with Annual Injury Prevalence

Annual injury prevalence differed significantly by country, with Myanmar and Sri Lanka having significantly lower prevalence rates than Thailand and Indonesia. A similar country pattern was identified for specific injuries, for fall injuries Myanmar and Thailand were the lowest, for sports injuries Myanmar the lowest, for motor vehicle injuries Myanmar and Sri Lanka the lowest and for fighting injuries adolescents from Thailand were the highest. Boys had higher annual injury prevalence rates than girls which was true for the different types of injuries. Substance use (current smoking and drinking alcohol) and greater psychological distress were found to be associated with annual injury prevalence rates. The highest influence of psychological distress was found with fighting injuries. Hunger as an indicator for low economic status and overweight were not found to be associated with annual injury prevalence nor with any specific injury (see Table 3).

### 3.3. Outcome Measures: Injury

For the main outcome, study participants were asked, “During the past 12 months, how many times were you seriously injured?” (serious injury was defined as when it makes you miss at least one full day of usual activities (such as school, sports, or a job) or requires treatment by a doctor or nurse). Eight options were provided, ranging from 1 = 0 times to 8 = 12 or more times. A response of “0” was described as not having sustained a serious injury, while a response of one or more times was classified as having experienced a serious injury. Additional items on injury included close-ended questions that addressed activity (During the past 12 months, what were you doing when the most serious injury happened?), external cause (During the past 12 months, what was the major cause of the most serious injury that happened to you?), how it happened (During the past 12 months, how did the most serious injury happen to you?), and type of injury (During the past 12 months, what was the most serious injury that happened to you?) (Response options see Table 1).

*Hunger*: A measure of hunger was derived from a question reporting the frequency that a young person went hungry because there was not enough food at home in the past 30 days (response options were from 1 = never to 5 = always) (coded 1 = most of the time or always and 0 = never, rarely or sometimes).

*Substance use variables*: Smoking cigarettes: During the past 30 days, on how many days did you smoke cigarettes? (Response options were from 1 = 0 days to 7 = all 30 days) (Coded 1 = 1 or 2 to all 30 days, and 0 = 0 days). Alcohol use: during the past 30 days, on how many days did you have at least one drink containing alcohol. Response options were from 1 = 0 days to 7 = all 30 days; Coded 1 = 1 or 2 to all 30 days, and 0 = 0 days. Drugs: During your life, how many times have you used drugs, such as glue, benzene, marijuana, cocaine, or mandrax? Response options were from 1 = 0 times to 4 = 10 or more times; Coded 1 = 1 or 2 to 10 or more times, and 0 = 0 times.

*Psychological distress*: Psychological distress was assessed with 5 items. Loneliness: “During the past 12 months, how often have you felt lonely?” (Response options were from 1 = never to 5 = always) (Coded 1 = most of the time or always and 0 = never, rarely or sometimes). Suicide ideation: “During the past 12 months, did you ever seriously consider attempting suicide?” (Response option was 1 = yes and 2 = no, coded 1 = 1, 2 = 0). No close friends:”How many close friends do you have?” (Response options 1 = 0 to 4 = 3 or more, coded 1 = 1, 2–4 = 0.). Anxiety or worried: During the past 12 months, how often have you been so worried about something that you could not sleep at night? (Response options were from 1 = never to 5 = always) (Coded 1 = most of the time or always and 0 = never, rarely or sometimes). Sadness: During the past 12 months, did you ever feel so sad or hopeless almost every day for two weeks or more in a row that you stopped doing your usual activities? (Response option 1 = yes and 2 = no) (Coded 1 = 1, 2 = 0). A psychological index was created by adding up all 5 items, and recoding the sum into low = no psychological distress, medium = 1 item of psychological distress and high = 2 or more psychological distresses endorsed.

### 3.4. Data Analysis

In order to compare study samples across countries each country sample was restricted to the age group 13 to 15 years inclusive, younger and older participants were excluded from the analyses. Data analysis was performed using STATA software version 10.0 (Stata Corporation, College Station, TX, USA). This software has the advantage of directly including robust standard errors that account for the sampling design, *i.e.*, cluster sampling owing to the sampling of school classes. In further analysis, the injury risk variable was recoded into two categories: not injured (0); injured at least once (1). Associations between potential risk factors and injuries among school children were evaluated calculating odds ratios (OR). Logistic regression was used for evaluation of the impact of explanatory variables on risk for injury (binary dependent variable). The dependent variable was the injury event, and the independent variables were factors which significantly increased injury risk in the univariate analysis. For the individual risk behaviour analyses, crude and adjusted odds ratios (ORs) and associated 95% confidence intervals were calculated for each level of exposure.

In the analysis, weighted percentages are reported. The reported sample size refers to the sample that was asked the target question. The two-sided 95% confidence intervals are reported. The *P*-value less or equal to 5% is used to indicate statistical significance. Both the reported 95% confidence intervals and the *P*-value are adjusted for the multi-stage stratified cluster sample design of the study.

## 4. Discussion and Study Limitations

In this study of in-school adolescents in four Southeast Asian countries using the Global School Health Survey of 2007/2008, a high percentage of adolescents (44.2%) reporting one or more serious injuries within the past 12 months was found, ranging from 27.0% in Myanmar to 46.8% in Thailand, and it has been significantly more often in boys (50.5%) than girls (34.3%) in all countries. This annual prevalence of severe injury was similar to that found in some other studies, South African Grade 8 students (52% among boys and 33% among girls) [24], Lithuanian school children (59% among boys and 40% among girls) [14], among 11-, 13- and 15-year old youth in 11 countries 41.3% [5], and among 35 countries between 33% and 62% across countries among males (19% to 39% among females) [12], and Scottish school children 41.9% [25] of all children were injured and needed medical treatment in the past 12 months. However, it was lower than found in a previous local study among school children in Thailand (66%) [6] and among school children in six African countries (68.2%) [4]. The annual injury prevalence rates found in this Southeast Asian sample may still be an underestimate considering a decline of estimates over a 12 month recall period. Mock *et al*. [26] found in a Ghanaian setting that longer recall periods significantly underestimate the injury rate compared to shorter recall periods. A possible explanation for the differences between injury rates in the different study countries may be due to differences in exposure to injury risk, e.g., traffic load on the roads, access to vehicles, access to sport opportunities, *etc*. More research is needed to understand the differences in injury risk between countries. Regarding the type of injury, the highest annual prevalence rate in this study was found for falls (14.6%), sport (9.9%) and vehicle accident-related injuries (6.1%); also other studies report that these three were the most common activities associated with injury [10,27,28]. This analysis represents one of the first Southeast Asian cross national examinations of adolescent injury patterns. This study found large cross national variations in severe injury prevalence. It is not clear whether these variations are attributable to underlying differences in risk. The Myanmar sample had the lowest annual injury prevalence and Thailand the highest. Depending on the country the GSHS was administered at different times of the year. Risks for adolescent injury vary by season, and injuries are more reliably reported within three than 12 months [29]. Variations in the timing of the survey across countries may have impacted injury rates and hence the cross national comparisons [6]. In a multivariable regression analysis substance use (smoking and drinking) and mental distress were associated with injuries. Similar associations between risk behaviours and the occurrence of injury were found in other studies, e.g., substance use (smoking, drinking) and psychological distress [13,14,15,16,17].Variations in the strength and direction of associations were observed for different combinations of social risk factors and types of injury. The highest influence of psychological distress was found with fighting injuries. Contrary to other studies, hunger as an indicator for low economic status and overweight were not found to be associated with annual injury prevalence nor with any specific injury [12,18,19]. This study found that the observed risk for all injuries increased with the increasing number of psychological distresses and other risk behaviours (substance use). Gradients in risk for adolescent injury also found in other studies [4] indicates support for the targeting of multiple forms of risk behaviour simultaneously in health interventions [7]. There is also a need to consider an integrated approach to injury etiology in planning injury prevention and safety promotion activities among school children, paying particular attention to lifestyle factors, which have the potential to influence risk for injuries. Efforts have to be intensified to include the prevention of violence and injury, along with sexual and reproductive health, healthy lifestyles, mental health and mental well-being, in adolescent health programmes [8].

This study had several limitations. Firstly, the GSHS only enrolls adolescents who are in school. School-going adolescents may not be representative of all adolescents in a country as the occurrence of injury and injury related risk behaviour may differ between the two groups. As the questionnaire was self-completed, it is possible that some study participants may have mis-reported either intentionally or inadvertently on any of the questions asked. Intentional miss-reporting was probably minimised by the fact that study participants completed the questionnaires anonymously. Furthermore, this study was based on data collected in a cross sectional survey. We cannot, therefore, ascribe causality to any of the associated factors in the study. Finally, the analysis was limited to the risk factors included in the GSHS. There are some other potentially important risk and protective factors (e.g., over-activity, failure to use seatbelts and bicycle helmets, being the perpetrator of an aggressive/bullying behaviour, ongoing conflict with parents, urban/rural situation, family, school or material supports, supervised or unsupervised school areas) [16,27,30] that could be associated with the occurrence of injury that were not measured. Finally, the injury survey tool collects only information on the ‘most serious injury’ and therefore risks not reflecting the true burden of injuries in these communities if a large number of other injuries collectively cause a greater burden/distress/absence or have different aetiologies, consequences or associations.

## 5. Conclusions

In this study, a high annual injury prevalence was found among adolescents in four Southeast Asian countries. Risk behaviour including substance use (smoking and drinking) and mental distress were found to be associated with injuries. There is also a need to consider an integrated approach to injury etiology in planning injury prevention and safety promotion activities among school children, paying particular attention to lifestyle factors, which have the potential to influence risk for injuries. Efforts have to be intensified to include the prevention of violence and injury, along with sexual and reproductive health, healthy lifestyles, mental health and mental well-being, in adolescent health programmes [8].

## References

[1] Hyder A.A., Puvanachandra P., Tran N.H. (2008). Child and adolescent injuries: A new agenda for child health. Inj. Prev..

[2] WHO/UNICEF (2005). Child and Adolescent Injury Prevention: A Global Call to Action.

[3] Hyder A.A., Labinjo M., Muzaffar S.S.F. (2006). A new challenge to child and adolescent survival in urban Africa: An increasing burden of road traffic injuries. Traffic Inj. Prev..

[4] Peltzer K. (2008). Injury and social determinants among in-school adolescents in six African countries. Inj. Prev..

[5] Molcho M., Harel Y., Pickett W. (2006). The epidemiology of non-fatal injuries among 11-, 13- and 15-year old youth in 11 countries: Findings from the 1998 WHO-HBSC cross-national survey. Int. J. Inj. Contr. Saf. Promot..

[6] Kozik C.A., Suntayakorn S., Vaughn D.W., Suntayakorn C., Snitbhan R., Innis B.L. (1999). Causes of death and unintentional injury among schoolchildren in Thailand. Southeast Asian J. Trop. Med. Publ. Health.

[7] Patton G.C., Coffey C., Sawyer S.M., Viner R.M., Haller D.M., Bose K., Vos T., Ferguson J., Mathers C.D. (2009). Global patterns of mortality in young people: A systematic analysis of population health data. Lancet.

[8] Plianbangchang S. (2011). Promoting adolescent health and development in South-East Asia. Indian J.Community Med..

[9] Pant P.R., Towner E. (2010). Systematic review of community-based studies of unintentional injuries in children in south east Asian countries. Inj. Prev..

[10] WHO (2010). Profile of Child Injuries: Selected Member States in the Asia-Pacific Region.

[11] Navaratne K.V., Fonseka P., Rajapakshe L., Somatunga L., Ameratunga S., Ivers R., Dandona R. (2009). Population-based estimates of injuries in Sri Lanka. Inj. Prev..

[12] Pickett W., Molcho M., Simpson K., Janssen I., Kuntsche E., Mazur J., Harel Y., Boyce W.F. (2005). Cross national study of injury and social determinants in adolescents. Inj. Prev..

[13] Pickett W., Schmid H., Boyce W.F., Simpson K., Scheidt P.C., Mazur J., Molcho M., King M.A., Godeau E., Overpeck M., Aszmann A., Szabo M., Harel Y. (2002). Multiple risk behavior and injury: An international study of youth in 12 countries. Arch. Pediatr. Adolesc. Med..

[14] Starkuniviene S., Zaborski A. (2005). Links between accidents and lifestyle factors among Lithuanian school children. Medicina (Kaunas).

[15] Pickett W., Garner M.J., Boyce W.F., King M.A. (2002). Gradients in risk for youth injury associated with multiple-risk behaviours: A study of 11,329 Canadian adolescents. Soc. Sci.Med..

[16] Mytton J., Towner E., Brussoni M., Gray S. (2009). Unintentional injuries in school-aged children and adolescents: Lessons from a systematic review of cohort studies. Inj.Prev..

[17] Muula A.S., Siziya S., Rudatsikira E. (2011). Prevalence and socio-demographic correlates for serious injury among adolescents participating in the Djibouti 2007 Global School-based Health Survey. BMC Res. Notes.

[18] Bazelmans C., Coppieters Y., Godin I., Parent F., Berghmans L., Dramaix M., Levêque A. (2004). Is obesity associated with injuries among young people?. Eur. J. Epidemiol..

[19] Simpson K., Janssen I., Craig W.M., Pickett W. (2005). Multilevel analysis of associations between socioeconomic status and injury among Canadian adolescents. J. Epidemiol. Community Health.

[20] CDC The Global School and Health Survey Background.

[21] Becker A.E., Roberts A.L., Perloe A., Bainivualiku A., Richards L.K., Gilman S.E., Striegel-Moore R.H. (2010). Youth health-risk behavior assessment in Fiji: The reliability of Global School-based Student Health Survey content adapted for ethnic Fijian girls. Ethn. Health.

[22] World Health Organization (WHO) (2011). World Health Statistics.

[23] UNICEF Myanmar: Statistics.

[24] Peltzer K. (2006). Injury and lifestyle factors among school-aged Black and White South African children in the Limpopo Province. Afri. Saf. Promot..

[25] Currie C.E., Williams J.M., Wright P., Beattie T., Harel Y. (1996). Incidence and distribution of injury among schoolchildren aged 11-15. Inj. Prev..

[26] Mock C.N., Acheampong F., Adjei S., Koepsell T. (1999). The effect of recall on estimation of incidence rates for injury in Ghana. Int. J. Epidemiol..

[27] Pickett W., Dostaler S., Craig W., Janssen I., Simpson K., Shelley S.D., Boyce W.F. (2006). Associations between risk behavior and injury and the protective roles of social environments: An analysis of 7,235 Canadian school children. Inj.Prev..

[28] Williams J.M., Wright P., Currie C.E., Beattie T.F. (1998). Sports related injuries in Scottish adolescents aged 11-15. Br. J. Sports Med..

[29] Harel Y., Overpeck M.D., Jones D.H., Scheidt P.C., Bijur P.E., Trumble A.C., Anderson J. (1994). The effect of recall on estimating annual nonfatal injury rates for children and adolescents. Am. J. Public Health.

[30] Jiang X., Li D., Boyce W., Pickett W. (2008). Alcohol consumption and injury among Canadian adolescents: Variations by urban-rural geographic status. J.Rural Health.

